# Recent Novel High-Tech Researches in Molecular Biology

**DOI:** 10.1155/2015/749160

**Published:** 2015-01-28

**Authors:** Calvin Yu-Chian Chen, Kuo-Chen Chou, James David Adams, Tai-Ping Fan, Gerhard Litscher

**Affiliations:** ^1^Human Genetic Center, Department of Medical Research, China Medical University Hospital, Taichung 40447, Taiwan; ^2^Research Center for Chinese Medicine & Acupuncture, China Medical University, Taichung 40402, Taiwan; ^3^School of Medicine, College of Medicine, China Medical University, Taichung 40402, Taiwan; ^4^Department of Biomedical Informatics, Asia University, Taichung 41354, Taiwan; ^5^Gordon Life Science Institute, Boston, MA 02478, USA; ^6^Center of Excellence in Genomic Medicine Research (CEGMR), King Abdulaziz University, Jeddah 21589, Saudi Arabia; ^7^School of Pharmacy, University of Southern California, Los Angeles, CA 90033, USA; ^8^Department of Pharmacology, University of Cambridge, Cambridge, UK; ^9^Research Unit of Biomedical Engineering in Anesthesia and Intensive Care Medicine, Research Unit for Complementary and Integrative Laser Medicine, and TCM Research Center Graz, Medical University of Graz, 8036 Graz, Austria

In this special issue recent novel high-tech research in molecular biology is discussed. Since the fast deployment in physics, chemistry, mathematics, and computer science, the recent molecular biology research becomes an evidence-based study even to a single molecular-level study. Thus we call this special issue for novel modern technology including next-generation sequencing methods, proteomics, bioinformatics, genomics, and computational systems biology.

S. El Shamieh et al. reported ophthalmic and genetic findings in families with autosomal recessive rod-cone dystrophy (arRCD) and RP1 mutations. Genomic DNA was investigated using a customized next-generation sequencing panel targeting up to 123 genes implicated in inherited retinal disorders. Sequencing identified 9 RP1 mutations in 7 index cases. Eight of the mutations were novel. Among these mutations, 4 belong to a region previously associated with arRCD and 5 others in a region previously associated with adRCD. Interestingly, a prevalence of ≈2.5% points out the necessity of sequencing RP1 in sporadic and recessive cases of RCD. The authors pointed out that further functional studies would strengthen our knowledge in the physiology of retinal photoreceptors.

The topic of H. Ohashi et al. was “*Next-generation technologies for multiomics approaches including interactome sequencing*.” They outlined a variety of new innovative techniques and discussed their use in omics research (e.g., genomics, transcriptomics, metabolomics, proteomics, and interactomics). The possible applications of these methods in future medical and life science research were also discussed, including an interactome-sequencing technology, developed by the authors.

J. Guo et al. investigated a virus-resistant transgenic sugarcane involving coat protein gene silencing by RNA interference (RNAi), which is a novel strategy for producing viral resistant plants. It can lead to target gene silencing, thus suppressing target gene expression. In this study, the conserved region of coat protein (CP) genes was selected as the target gene. The genetically modified sorghum mosaic virus-resistant lines of cultivar ROC22 provide resistant germplasm for breeding lines and can also serve as resistant lines having the same genetic background for study of resistance mechanisms.

The paper by T. Al-Edani et al. deals with the influence of female aging on human cumulus cells (CCs) genes. There was a need for an extensive analysis of age impact on transcriptome profile to link oocyte quality and developmental potential with patient's age. CCs from patients of three age groups were analyzed individually using whole genome U133 Plus 2.0 GeneChip Affymetrix microarrays. The authors focused on pathways affected by aging in CCs that may explain the decline of oocyte quality with age. Specific molecular signatures were characterized for the three age categories. It was revealed that the pathways impacted by age were potential targets of specific microRNAs previously identified in CCs small RNAs sequencing.

V. Ambriz-Aviña et al. applied flow cytometry (FCM) to characterize bacterial physiological responses. They reviewed how FCM has been applied to characterize distinct physiological conditions in bacteria including responses to antibiotics and other cytotoxic chemicals and physical factors. Since FCM is suitable for performing studies at the single-cell level, the authors were able to describe how this powerful technique has yielded invaluable information about the heterogeneous distribution of differently and even specialized responding cells and how it may help to provide insights about how cell interaction takes place in complex structures, such as those that prevail in bacterial biofilms.

In the project introduced by Y. Cai et al., a highly precise quantitative method based on the digital polymerase chain reaction (dPCR) technique was developed to determine the weight of pork and chicken in meat products. Currently, real-time quantitative polymerase chain reaction (qPCR) is used for quantitative molecular analysis of the presence of species-specific DNAs in meat products, but it is limited in several aspects. By using the dPCR method, the authors found that the relationships between the raw meat weight and DNA weight and between the DNA weight and DNA copy number were both close to linear. This enabled them to establish formulae to calculate the raw meat weight based on the DNA copy number. The accuracy was verified using samples of pork and chicken powder mixed in known proportions. Quantitative analysis indicated that dPCR is highly precise and therefore has the potential to be used in routine analysis by government regulators and quality control departments, once some technical flaws have been resolved.

G. N. Sundell and Y. Ivarsson investigated interaction analysis through proteomic phage display, which is a powerful technique for profiling specificities of peptide-binding domains. Using highly diverse combinatorial peptide phage libraries, the method is suited for the identification of high-affinity ligands with inhibitor potential. A complementary but considerably less explored approach is to display expression products from genomic DNA, cDNA, open reading frames (ORFs) or from microarray oligonucleotide libraries designed to encode for defined regions of a target proteome are displayed on phage particles. This review focused on the use of proteomic phage display to uncover protein-protein interactions of potential relevance for cellular function. The method is particularly suited for the discovery of interactions between peptide-binding domains and their targets. The authors discussed the largely unexplored potential of this method in the discovery of domain-motif interactions of potential biological relevance.

Z.-L. Lai et al. contributed a paper entitled “*Methylation-associated gene silencing of RARB in areca carcinogens induced mouse oral squamous cell carcinoma.*” DNA methylation is a major epigenetic alternation of genome that regulates this crucial aspect of its function without changes in the DNA sequence. It is also thought to play an important role in carcinogenesis. Regarding oral squamous cell carcinoma (OSCC) development, chewing areca is known to be a strong risk factor in many Asian cultures. Therefore, the authors established an OSCC induced mouse model by 4-nitroquinoline-1-oxide (4-NQO), or arecoline, or both treatments, respectively. These are the main two components of the areca nut that could increase the occurrence of OSCC. The effects were examined with the noncommercial MCGI (mouse CpG islands) microarray for genome wide screening of the DNA methylation aberrant in induced OSCC mice. The results showed that retinoic acid receptor b (RARB) was indicated in hypermethylation at the promoter region and the loss of expression during cancer development. According to the results of real-time PCR, it was shown that de novo DNA methyltransferases were involved in gene epigenetic alternations of OSCC. Collectively, the results showed that RARB hypermethylation was involved in the areca-associated oral carcinogenesis.

W. Eilers et al. explored to which extent isoforms of the regulator of excitation-contraction and excitation-transcription coupling, calcium/calmodulin protein kinase II (CaMKII), contribute to the specificity of myocellular calcium sensing between muscle types and whether concentration transients in its autophosphorylation can be simulated. Qualitative differences existed between fast (gastrocnemius medialis) and slow type (muscle soleus) for the expression pattern of CaMKII isoforms. In silico assessment emphasized the importance of mitochondrial calcium buffer capacity for excitation-induced CaMKII autophosphorylation but did not predict its isoform specificity. The findings exposed that CaMKII autophosphorylation with paced contractions is regulated in an isoform and muscle type-specific fashion and highlight properties emerging for phenotype-specific regulation of CaMKII.

X. Li et al. introduced a novel open-source software (CELLCOUNTER) for counting cell migration and invasion in vitro. In contrast to the usually performed manual counting of cells in Transwell Boyden chamber based migration/invasion assays, this application is reported to be capable of recognizing and counting the total number of cells through an intuitive graphical user interface. The counting can be performed in batch, and the counting results can be visualized and further curated manually. The authors therefore conclude that the new software will be helpful in streamlining the experimental process and improving the reliability of the data acquisition.

In their paper, C.-J. Shen et al. investigated aberrant methylation in cloned porcine genome. Cloned animals usually exhibit defects in physical characteristics or aberrant epigenetic reprogramming, especially in some important organ development, such as heart valve and bone retardation. Osteopontin (OPN) is an extracellular-matrix protein that is involved with heart and bone development and diseases. The authors investigated the correlation between OPN mRNA and its promoter methylation changes by the 5-aza-dc treatment in fibroblast cell and promoter assay. Data revealed that four methylated CpG sites presenting in the −2615 to −2239 bp region cause significant downregulation (approximately 75%) of OPN promoter activity. They also perform the protein-protein docking by software named Z-dock. Besides, the protein-protein complex also performed molecular dynamics simulation for validation. From all the evidences, they propose a novel mechanism and suggest that methylation in the OPN promoter plays a crucial role in the regulation of OPN expression that was found in cloned pigs genome.

The topic of Y.-A. Tsou et al.'s paper was “*Evaluation of correlation of cell cycle proteins and Ki-67 interaction in paranasal sinus inverted papilloma prognosis and squamous cell carcinoma transformation.*” The authors used protein expression patterns by immunohistochemical methods to see that the expression of p53, p16, p21, and p27 belongs to cell-cycle-regulators and PCNA, Ki-67 the proliferation markers in 60 inverted papilloma and 10 of them with squamous cell carcinoma transformation of the sinonasal tract. Significantly elevated levels of Ki67 and PCNA in IP with squamous cell carcinoma transformation of sinonasal tract compared with inverted papilloma were revealed. No variation of p16, p21, p27, and p53 expression was correlated to the IP malignant transformation. In conclusion, this is a first study that showed the correlation of Ki67 interacted with CDK1 and leads to malignant transformation and the elevated PLUNC expression in the sinonasal IPs with multiple recurrences in humans. They also employ the Z-Dock for protein-protein docking of related target proteins, CDK1 with target proteins (a) Ki-67, (b) p27, and (c) PCNA. From dihedrals angle of key binding residues and cluster analyses of all CDK1 and the binding proteins, they propose a novel mechanism of Ki-67, p27, and PCNA in cell cycle.

K.-B. Chen et al. compared the use of traditional Chinese medicine (TCM) against pregnane X receptor in the treatment of cardiovascular disease. The human pregnane X receptor, PXR, plays a crucial role in exogenous and endobiotic metabolism for rabbits, rats, mice, and humans. PXR activation can protect the blood vessels from damage caused by hazardous substances. The authors aimed to investigate the potent lead compounds as PXR receptor agonist against cardiovascular disease. To improve drug development of TCM compounds, they also aimed to investigate the potent lead compounds as PXR agonists from the TCM compounds in TCM Database@Taiwan. The top three TCM compounds, BEMG, Ixerisoside, and Tangshenoside II, displayed higher potent binding affinities than the positive control, PNU-142721, in the docking simulation. They also perform the very time-consuming molecular dynamics simulation for validation of the stability of these potent compounds binding with PXR protein. Hence, the authors propose BEMG and Tangshenoside II, as potential lead compounds for further study in drug development process with the PXR protein.

